# Decoding Glioblastoma Through Liquid Biopsy: Molecular Insights and Clinical Prospects

**DOI:** 10.3390/cells15030309

**Published:** 2026-02-06

**Authors:** Tomasz Wasiak, Maria Jaskólska, Kamil Filiks, Jakub Bartkowiak, Adrianna Rutkowska

**Affiliations:** 1 Department of Molecular Biology, Chair of Medical Biology, Medical University of Lodz, Zeligowskiego 7/9 St., 90-752 Lodz, Poland; maria.jaskolska@stud.umed.lodz.pl (M.J.); kamil.filiks@student.umed.lodz.pl (K.F.); jakub.bartkowiak@student.umed.lodz.pl (J.B.); adrianna.rutkowska@umed.lodz.pl (A.R.); 2 Department of Research and Development, LEK-AM Pharmaceutical Company Ltd., Inwestycyjna 7 St., 95-050 Konstantynow Lodzki, Poland

**Keywords:** liquid biopsy, glioblastoma, cell-free nucleic acids, extracellular vesicles, circulating tumor cells, non-invasive diagnostics, tumor heterogeneity

## Abstract

**Highlights:**

**What are the main findings?**

- Extracellular vesicles, ctDNA, circulating RNA species, and CTCs provide complementary molecular information that reflects glioblastoma heterogeneity and treatment dynamics.
- CSF-derived biomarkers show consistently higher sensitivity than plasma biomarkers, while EV-associated signatures strongly correlate with tumor biology, therapy resistance, and immune evasion.

**What are the implications of the main findings?**

- Liquid biopsy can support diagnosis, early detection of recurrence and differentiation between progression and pseudoprogression when integrated with neuroimaging.
- Standardized multi-marker assays combining ctDNA, EV-RNA (Extracellular Vesicle RNA) and circulating RNA profiling may accelerate the translation of liquid biopsy into routine glioblastoma management.

**Abstract:**

Liquid biopsy (LB) offers a minimally invasive approach to characterizing and monitoring glioblastoma (GB), a tumor marked by extensive heterogeneity, limited surgical accessibility and rapid molecular evolution. By analyzing circulating tumor-derived components such as circulating tumor DNA (ctDNA), extracellular vesicles (EVs), circulating RNA species and circulating tumor cells (CTC), LB provides dynamic molecular information that cannot be captured by neuroimaging or single-site tissue sampling. Cerebrospinal fluid (CSF) currently yields the highest sensitivity for detecting tumor-specific alterations, while plasma enables repeat monitoring despite lower biomarker abundance. EVs have gained particular prominence due to their ability to preserve DNA, RNA, and protein cargo that reflects key genomic changes, treatment resistance mechanisms, and immune evasion. Although advances are substantial, clinical implementation remains constrained by low analyte concentrations, methodological variability, limited standardization and the high cost of testing, which is rarely reimbursed by insurers. This review summarizes current evidence on circulating biomarkers in GB and highlights research priorities essential for integrating LB into future diagnostic and therapeutic workflows.

## 1. Introduction and Clinical Background

Glioblastoma (GB) is the most aggressive primary brain tumor, accounting for approximately 15% of primary brain and CNS tumors, with an annual incidence of 3.2 per 100,000 and a median survival of 12–15 months despite multimodal therapy [1,2,3]. Neuroimaging is the standard for detection and therapy monitoring [4], while tissue biopsy is invasive and inherently limits repeated sampling. Liquid biopsy (LB) offers a minimally invasive, repeatable approach that may enable longitudinal assessment of disease activity and treatment response, analogous to serial biomarker monitoring in other cancers, and may support more timely therapeutic decisions. Given the infiltrative growth pattern and marked intratumoral heterogeneity of GB, single tissue samples may not fully represent evolving molecular profiles, particularly under treatment pressure [5,6]. In this review, we focus on the significant classes of circulating analytes: circulating tumor DNA (ctDNA), circulating RNA species, extracellular vesicles (EVs), and circulating tumor cells (CTCs), and evaluate their diagnostic, prognostic, and therapeutic relevance in GB, with emphasis on current limitations and priorities for clinical translation.

## 2. Unmet Clinical Needs in Glioblastoma Diagnosis

Despite advances in neuroimaging, neurosurgery and systemic therapy, GB remains a cancer with an inferior prognosis, with median survival measured in months rather than years. Several studies emphasize that one of the main reasons for this is significant gaps in the diagnosis and monitoring of the disease, which current clinical tools (tissue biopsy, MRI) are unable to fully address [7,8,9]. Below is a summary of key unmet clinical needs repeatedly mentioned in the literature, where LB is seen as a potential supplement or future alternative to traditional methods.

### 2.1. Early, Non-Invasive Diagnosis and Preoperative Stratification

In a many of patients, tumor location limits the safe acquisition of representative tissue for histopathological and molecular analysis. At present, there are no widely implemented non-invasive blood- or cerebrospinal fluid (CSF)-based assays enabling reliable diagnosis and molecular classification of GB [8,10]. An optimal non-invasive approach would need to capture key diagnostic and predictive alterations, including *IDH* and H3 mutations, *EGFR* amplification, +7/−10, *TERT* promoter mutations, as well as treatment-relevant markers such as *MGMT* promoter methylation, EGFRvIII expression, and *PD-L1* status, which are currently available primarily from tissue-based analyses [11,12,13,14].

### 2.2. Differentiating True Progression from Pseudoprogression and Radionecrosis

A well-recognized clinical challenge in GB management is the inability to reliably distinguish true tumor progression (TP) from pseudoprogression (PsP) and treatment-related radionecrosis using standard MRI. Chemo-radiotherapy, particularly temozolomide (TMZ) combined with radiotherapy, may induce post-radiation necrosis and blood–brain barrier (BBB) disruption, leading to contrast enhancement that mimics tumor growth [15]. Conversely, antiangiogenic therapies can reduce MRI contrast signals by altering vascular permeability without reducing tumor burden [16]. Such effects may result in misinterpretation, premature treatment changes, or unnecessary surgical interventions [6,17]. Advanced neuroimaging techniques, including perfusion-weighted imaging (PWI), diffusion-weighted imaging (DWI), magnetic resonance spectroscopy (MRS), and positron emission tomography (PET), improve diagnostic accuracy but remain insufficient to replace biopsy or longitudinal follow-up [18,19,20]. LB may complement imaging by capturing molecular evolution, clonal selection, and emerging treatment resistance that are not visible on conventional magnetic resonance imaging (MRI) [8,21]. However, routine clinical implementation is limited by the lack of validated quantitative thresholds, decision-making algorithms, and integrated frameworks combining molecular and radiological data, and by the absence of consensus regarding biomarker-driven management decisions [6,7].

### 2.3. Monitoring Minimal Residual Disease and Early Detection of Recurrence

Post-treatment monitoring in GB is currently based on follow-up MRI at defined intervals, and recurrence is often detected only after macroscopic changes have occurred, limiting the effectiveness of subsequent therapies. Importantly, unlike in leukemia, no standardized marker for minimal residual disease (MRD) exists in GB [21,22,23]. LB has been proposed as a tool to detect early increases in ctDNA or selected miRNAs/EVs before progression becomes evident on MRI, to capture molecular dynamics during treatment (e.g., emergence or disappearance of specific clones), and to support evaluation of novel therapies in clinical trials. However, prospective multicenter studies demonstrating that LB-guided early intervention improves survival are still lacking [24].

### 2.4. Capturing the Spatial and Temporal Heterogeneity of the Tumor

GB is characterized by extreme genetic and phenotypic heterogeneity, both with single lesions and between secondary lesions. Numerous data show that a single paraffin block from a biopsy or resection often does not reflect the full spectrum of molecular changes, and performing subsequent biopsies poses a significant risk to the patient [25,26]. The literature emphasizes that classic brain tumor diagnostics most often provide only a “single snapshot” of the tumor at a specific point in time. At the same time, there is a lack of widely implemented tools for non-invasive, repeated tracking of clonal evolution under the influence of treatment (e.g., surgery, chemo-radiotherapy) [27,28]. In this context, LB, including the analysis of ctDNA, EVs, and CTCs, could theoretically better reflect the full spectrum of clones present in the tumor mass and in possible secondary foci in the central nervous system [29]. Nevertheless, the available data remain fragmentary and based mainly on small research cohorts and preliminary technical analyses [30].

### 2.5. Standardization, Validation, and Integration with Decision Algorithms

Current guidelines from international neuro-oncology societies do not recommend routine use of LB in patients with GB, mainly due to significant heterogeneity in pre-analytical procedures (type of tubes, time to processing, isolation method), diversity of analytical methods (ddPCR vs. various NGS panels, EV/CTC isolation), and research limitations including small, single-center cohorts, lack of external validation of proposed biomarker panels, and insufficient cost-effectiveness analysis [21,29,31,32].

## 3. The Biology of Liquid Biopsy

Cell-free DNA (cfDNA) is detectable in the circulation of healthy individuals, but elevated cfDNA levels have been associated with tumor burden since 2001 [33,34]. In healthy subjects, cfDNA concentrations typically range from 0 to 100 ng/mL, whereas cancer patients, including those with gliomas, often exhibit markedly higher levels, sometimes exceeding 1000 ng/mL [35]. CfDNA abundance varies by tumor type and anatomical location, with brain tumors releasing lower amounts into peripheral blood due to physiological barriers, which complicates detection compared with extracranial malignancies [36]. CfDNA originates primarily from apoptotic and necrotic tumor cells, as well as from lysed CTCs [33]. Methylation-based tissue-of-origin analyses indicate that most cfDNA in healthy individuals derives from hematopoietic cells, while in cancer patients, a substantial fraction originates from tumors [37,38,39]. Fragment size reflects the mode of release, with apoptotic cfDNA averaging ~167 bp and necrosis generating longer fragments exceeding 10 kbp [40,41]. CfDNA may also be encapsulated within extracellular vesicles, protecting longer DNA fragments from degradation, and increased exosome secretion has been reported in glioma cells [42,43]. Preanalytical factors, particularly blood collection methods, influence cfDNA yield, with plasma preferred over serum to minimize leukocyte-derived contamination [44].

EVs comprise a heterogeneous group of membrane-bound particles released by cells into the extracellular space and represent an important source of biologically informative material for LB. EVs are commonly classified into exosomes, microvesicles and apoptotic bodies, which differ in their biogenesis, size and biological roles. These vesicles carry proteins, lipids and nucleic acids that reflect the molecular state of the cell of origin, making them particularly relevant as tumor-derived biomarkers [45,46]. Exosomes are the smallest EVs (approximately 40–200 nm) and originate from the endosomal system through the formation of multivesicular bodies and their fusion with the plasma membrane. Their molecular cargo includes proteins, mRNA, microRNA and DNA fragments, and mirrors key features of tumor biology. Exosome release is regulated by cellular stress conditions such as hypoxia, inflammation and metabolic stress, all of which are characteristic of the GB microenvironment. Through intercellular transfer of bioactive molecules, exosomes participate in tumor progression, immune modulation and angiogenesis EVs [45,47,48]. Microvesicles are larger EVs (approximately 100–1000 nm) formed by direct outward budding of the plasma membrane. Their release is driven by cytoskeletal reorganization, membrane lipid redistribution and calcium-dependent enzymatic activity [45,49]. Microvesicles transport membrane receptors, signaling molecules and nucleic acids and have been implicated in tumor invasion, therapy resistance and remodeling of the tumor microenvironment. Their secretion is enhanced by cellular activation, inflammatory signals and mechanical stress, conditions frequently present in malignant tumor EVs [50,51]. Apoptotic bodies represent the largest EV population (approximately 500 nm to 5 µm) and are released during programmed cell death [45,52]. Although initially regarded as inert cellular debris, apoptotic bodies are now recognized as active participants in immune regulation and intercellular communication through the transfer of nucleic acids, proteins and cellular organelles. In cancer, increased apoptosis contributes to their presence in circulation, further expanding the spectrum of EV-associated tumor signals accessible by LB EVs [53,54].

CTCs are intact cancer cells shed into the bloodstream from primary or metastatic sites. Their rarity, approximately one CTC per billion blood cells, necessitates sensitive isolation techniques based on size exclusion, antigen expression, or microfluidic enrichment [55,56]. Upon entering circulation, CTCs face immune surveillance, particularly from NK cells, but evade destruction through interactions with activated platelets. Platelets cloak CTCs, exchanging surface antigens such as major histocompatibility complex (MHC) molecules, and secrete immunosuppressive factors, including vascular endothelial growth factor (VEGF) and transforming growth factor-beta (TGF-β), which impair dendritic cell maturation and downregulate NK cell-activating receptors [57,58,59,60].

The biological behavior of GB and the integrity of the BBB critically influence the performance of liquid biopsy. BBB restriction limits the release of tumor-derived nucleic acids and vesicles into peripheral blood, resulting in low plasma ctDNA and EV concentrations and reduced sensitivity compared with systemic cancers [60,61,62]. In contrast, CSF represents a tumor-proximal compartment in which ctDNA and EVs accumulate at higher variant allele fractions and more accurately reflect tumor heterogeneity. CSF-based analyses have demonstrated superior detection of clinically relevant alterations, including *EGFR* amplification, *IDH1/2* mutations, *TP53* variants, and *CDKN2A/B* deletions [60,63,64]. Although plasma is preferred for longitudinal monitoring due to its minimal invasiveness, its diagnostic utility remains constrained by BBB physiology [61,62,65]. Consequently, CSF-based liquid biopsy is the most informative approach in GB when clinically feasible and safe. Detection is further challenged by the short circulation times of biomarkers: CTCs persist for 1–2.5 h, EVs for minutes, ctDNA for up to 2.5 h, and microRNAs for approximately 16.5 h [66,67,68,69].

## 4. Biofluid Sources: CSF, Blood, Tumor-Proximal Blood, Urine

**Figure 1:**
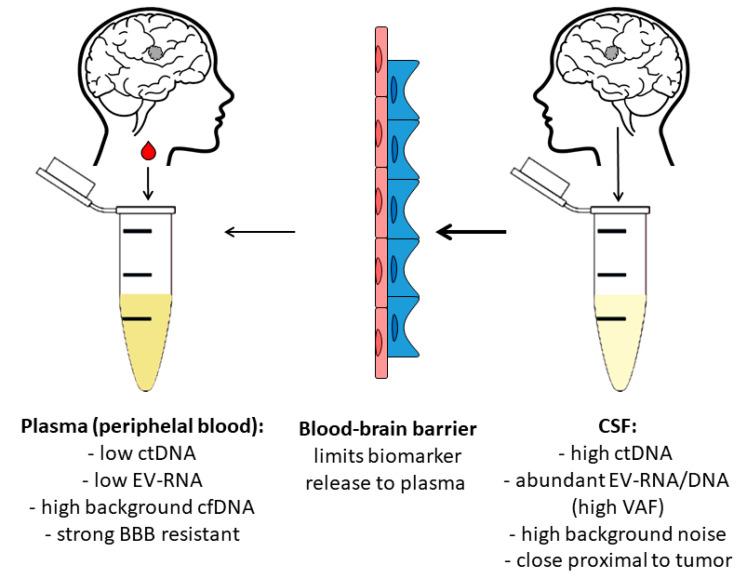
Relative abundance of tumor-derived biomarkers in plasma versus CSF in glioblastoma. CSF obtained intraoperatively from a subarachnoid leak contains higher level of tumor-derived material, including circulating tumor DNA (ctDNA), extracellular vesicles (EVs) and RNA, owing to its direct proximity to the tumor and minimal dilution. In contrast, plasma exhibits low biomarker abundance due to the substantial restrictions imposed by the blood-brain barrier (BBB) and the high background cell free DNA (cfDNA). This difference underpins the higher diagnostic sensitivity of CSF-based LB compared with peripheral blood-based liquid biopsy (LB). Plasma refers to peripheral blood-derived plasma and does not originate directly from the brain parenchyma. Colors differentiate plasma and CSF samples, while arrow thickness reflects the relative abundance of recoverable tumor-derived biomarkers.

LB for GB requires careful consideration of the biofluid source, as each compartment offers distinct advantages and limitations in reflecting tumor biology. CSF is anatomically closest to the tumor microenvironment, and multiple recent studies demonstrate significantly higher detection rates of tumor-derived nucleic acids and EVs in CSF than in plasma (e.g., >60% in CSF vs. ~30–40% in plasma) [8,23,70]. This superior sensitivity is attributed to minimal dilution, reduced background cfDNA and direct shedding of tumor material into CSF. In contrast, plasma remains the most accessible, minimally invasive sample type, making it attractive for longitudinal monitoring. However, it suffers from low variant allele fractions and high background noise owing to the BBB and systemic dilution (Figure 1) [4,9].

New research efforts also explore tumor-proximal blood (e.g., venous drainage from tumor-fed vessels) and alternative fluids, such as urine; however, these approaches remain in early stages of investigation. From a clinical perspective, sampling of tumor-proximal blood is procedurally complex and has not demonstrated a clear advantage over established neurosurgical procedures for obtaining tissue for conventional histopathological analysis, thereby limiting its current applicability for routine or longitudinal clinical use [14,71,72] (Table 1). From a clinical perspective, CSF-based sampling is the most informative for genotyping and profiling tumor evolution in GB. In contrast, plasma offers practical utility for repeat sampling, albeit with recognition of its sensitivity limitations. Thus, in constructing an LB workflow for GB, authors should emphasize the trade-off between invasiveness and molecular yield, and clearly justify the choice of biofluid in relation to the clinical question, sampling interval and assay sensitivity. Despite its promise, lumbar puncture, the sole method used to obtain CSF, is contraindicated in patients with elevated intracranial pressure, limiting its widespread clinical application [73].

## 5. Markers for Liquid Biopsy

The concept of LB in GB is based on the detection of tumor-derived molecular markers in circulating biofluids that reflect dynamic tumor biology. These analytes capture key aspects of tumor behavior, including genetic and epigenetic alterations, transcriptional activity, cellular turnover, and treatment resistance. In contrast to tissue-based diagnostics, which provide a static, spatially limited snapshot, circulating biomarkers enable minimally invasive, repeatable assessment of tumor evolution over time. The most extensively studied markers include ctDNA, circulating RNA species, EVs carrying nucleic acids, and CSCs. Each marker differs in abundance, stability, analytical detectability, and clinical relevance, depending on tumor biology, biofluid characteristics, and BBB permeability. The following subsections summarize the biological basis, methodological considerations, and current clinical evidence for each LB marker category in GB.

### 5.1. Circulating Tumor DNA and Methylation Markers

CtDNA represents the fraction of cfDNA derived from tumor cells and carries tumor-specific genetic and epigenetic alterations, including somatic mutations, copy-number variations, and DNA methylation patterns. CtDNA is released into circulation through apoptosis, necrosis, and active secretion and typically constitutes a low-abundance, highly fragmented component of total cfDNA [74]. Among circulating biomarkers in glioma patients, cfDNA and cfRNA have been most extensively studied. Next-generation sequencing studies (NGS) report ctDNA detection rates of approximately 50% in advanced GB, depending on tumor stage and histopathological subtype [60,64]. Recurrent plasma ctDNA alterations include mutations in *TP53*, *EGFR*, *MET*, *PIK3CA*, and *NOTCH1*, as well as alterations in *NF1*, *APC*, *PDGFRA*, and amplifications of *ERBB2*, *MET*, and *EGFR* [60,75].

Beyond genomic alterations, epigenetic markers provide clinically relevant information. *MGMT* promoter methylation remains the most established epigenetic marker in gliomas, with blood-based assays demonstrating 51% sensitivity and 100% specificity for glioma detection [76]. However, BBB physiology limits plasma ctDNA detection, making CSF a more informative source. Detection rates of *MGMT* promoter methylation are higher in CSF than in serum (33.3% vs. 21.3%) [77]. CSF ctDNA profiles also show concordance with tumor tissue alterations, including *TP53* and *IDH1* mutations, *CDKN2A/B* deletions, and *EGFR* and *KDR* overexpression, and correlate with tumor progression in a substantial proportion of GB patients [55].

### 5.2. Circulating RNAs

Circulating RNA constitutes a diverse and biologically informative class of LB biomarkers in GB. Unlike ctDNA, which primarily reflects genomic alterations and cell death, RNA-based analytes capture active transcriptional programs and provide insight into tumor signaling, phenotype, and treatment resistance. Multiple RNA species, including microRNAs (miRNAs), long non-coding RNAs (lncRNAs), and circular RNAs (circRNAs), are detectable in plasma, serum and CSF, either freely circulating or protected within extracellular vesicles [78]. MiRNAs regulate gene expression post-transcriptionally and are involved in tumor progression and therapy resistance [79]. MiR-21 is the most consistently reported GB-associated miRNA, detectable in serum and CSF, and its levels decrease after treatment [80,81,82]. Other miRNAs, including miR-182, miR-210, miR-221, miR-222, and miR-454, have been associated with aggressive tumor behavior and poorer survival, while reduced levels of miR-15b, miR-23a, miR-133a, miR-150, miR-197, miR-497, and miR-548b-5p are frequently observed in GB patients [83,84,85]. Circulating miRNA profiles are further influenced by patient age, tumor size, and anatomical location [86].

LncRNAs regulate oncogenic signaling, angiogenesis, and therapeutic resistance [87,88]. Overexpression of HOTAIR and reduced GAS5 expression have been linked to poor prognosis and increased recurrence risk in GB [89,90]. CircRNAs, which are covalently closed RNA molecules, may function as miRNA sponges or regulators of cell cycle and apoptosis. Several circRNAs, including circ_0001649, circ_0034642, circ_0074362, circ_ITCH, circHIPK3, and circCPA4, have been associated with tumor progression or diagnostic discrimination in GB [91,92,93,94,95].

Tumor-educated platelets (TEPs) represent an additional source of circulating RNA [96]. Through active interactions with tumor cells, platelets acquire tumor-derived RNA signatures reflecting disease biology [97,98]. Platelet-derived RNA profiles have been shown to discriminate between glioma patients and healthy controls and to correlate with tumor grade and progression [99]. Compared with plasma cfRNA, TEP-derived RNA offers higher yields, greater stability, and greater resistance to degradation, supporting its potential utility for diagnosis and longitudinal monitoring [96,100].

### 5.3. Extracellular Vesicles (EVs)

Extracellular Vesicles (EVs) are membrane-bound particles that transport nucleic acids, proteins and other bioactive molecules reflective of their cell of origin. In GB, EVs serve both as biomarkers and active mediators of tumor progression. Their heterogeneous cargo, including EV-DNA, mRNA, miR, lncRNA, circRNA, and proteins, mirrors key genetic alterations such as *EGFR* amplification, *PTEN* loss, *TP53* mutations, and *IDH*1 mutations, providing insight into intratumoral heterogeneity and clonal evolution [101,102,103]. Tumor-specific EV subpopulations, including Tenascin-C-positive EVs, are elevated in plasma from newly diagnosed and recurrent GB patients and show promise as disease-specific biomarkers [103]. EV-associated proteins such as Syndecan-1 (CD138) and defined EV-miR panels have demonstrated diagnostic potential and may support postoperative monitoring [102,104]. Functionally, EVs contribute to therapeutic resistance. EVs derived from TMZ-resistant GB cells transfer lncRNAs, such as SBF2-AS1, promoting resistance by modulating *MGMT* promoter methylation and stemness pathways. EV-associated circRNAs have also been implicated in radioresistance and altered DNA damage responses [105]. In addition, GB-derived EVs participate in immune evasion by displaying PD-L1, which suppresses T-cell activation and may impair responses to immunotherapies [106]. Selective enrichment of tumor-derived EV subpopulations improves detection of clinically relevant mutations and enhances signal-to-noise ratios compared with bulk cfDNA [103].

### 5.4. Circulating Tumor Cells

Circulating tumor cells are intact tumor cells shed into the bloodstream that retain molecular and phenotypic features of the primary tumor [107]. In GB, CTCs have been identified in a subset of patients, with approximately 21% expressing GFAP while lacking CD45 expression, and frequently harboring *EGFR* amplification or EGFRvIII mutations [62,108]. Surgical resection does not appear to alter CTC counts significantly, and correlations with disease burden remain inconsistent. CTCs can be isolated using leukocyte depletion strategies, such as magnetic separation with anti-CD16 and anti-CD45 antibodies, achieving approximately 40% efficiency [62]. Subsequent immunophenotyping enables classification into molecular GB subtypes and reveals stem cell-like properties, including SOX2, OCT4, and NANOG expression. These features are associated with resistance to chemotherapy and radiotherapy, suggesting a potential role for CTCs in disease progression and treatment failure [108].

### 5.5. Prognostic Relevance of Circulating Biomarkers

Accumulating evidence suggests that circulating tumor-derived biomarkers carry prognostic information in GB. A higher baseline level of cfDNA or detectable ctDNA in plasma or CSF has been associated with increased tumor burden and poorer survival [109,110]. Longitudinal changes are more informative than single measurements, with rising ctDNA levels, increasing variant allele frequency, or expanding copy-number alterations preceding radiological progression, while declining ctDNA levels correlate with treatment response [35,111]. Similar associations have been reported for EVs, where increased abundance or altered cargo correlates with aggressive disease behavior [102]. However, these relationships remain heterogeneous and are influenced by biological and technical factors, including BBB integrity, tumor heterogeneity, and assay sensitivity. Consequently, while circulating biomarkers show promising prognostic associations, they are not yet validated as independent survival markers and should be interpreted in conjunction with established clinical and radiological parameters [35,62].

Several studies have also demonstrated associations between circulating biomarkers and conventional tissue-based features. Adverse histopathological characteristics, including high cellularity, necrosis, and microvascular proliferation, as well as unfavorable immunohistochemical profiles such as high Ki-67 index or unmethylated *MGMT* promoter status, are associated with increased detectability of tumor-derived nucleic acids in circulation [112]. CtDNA alterations show partial concordance with tissue genomic profiles, including *EGFR* amplification and *TERT* promoter mutations [7,9,110]. Changes in circulating biomarkers during radiotherapy and alkylating chemotherapy parallel clinical and radiological responses, with persistent tumor-derived signals indicating resistance and earlier progression [15,113,114]. Nevertheless, due to biological and technical variability, circulating biomarkers should currently be interpreted as complementary to histopathological and immunohistochemical assessment rather than as independent prognostic tools [7,113].

## 6. Technological Platforms and Pre-Analytical Variables

LB has become an important tool in oncology and molecular diagnostics, enabling minimally invasive detection of tumor-derived biomarkers in body fluids. Current technological platforms range from highly sensitive molecular assays to advanced sequencing-based approaches, while the reliability of results critically depends on rigorous pre-analytical handling. This section provides an overview of the principal analytical technologies used in LB, including digital (droplet) PCR (ddPCR), NGS, and biophysical methods, as well as key pre-analytical and analytical factors that influence assay performance.

### 6.1. Digital (Droplet) PCR

Digital (Droplet) PCR remains one of the most sensitive methods for detecting rare variants in low-abundance material such as ctDNA or EV-associated RNA. Its main advantage is absolute quantification with minimal susceptibility to amplification bias, enabling detection of mutant allele fractions below 0.01% [115,116]. High analytical performance has been demonstrated for the detection of resistance mutations, including *EGFR* T790M and *BRAF* V600E, as well as for longitudinal monitoring during targeted and immune-based treatments [117,118,119,120]. Recent advances in automated microfluidic platforms and error-correction algorithms have further improved reproducibility and facilitated broader clinical implementation, particularly for early detection of disease progression and treatment failure [121,122,123].

### 6.2. Next-Generation Sequencing

Next-Generation Sequencing represents the central technology for comprehensive LB analysis, enabling simultaneous assessment of point mutations, copy number alterations, structural variants and DNA methylation patterns [124]. Targeted NGS panels incorporating molecular barcoding and hybrid capture achieve detection limits of approximately 0.1–0.2% while maintaining high analytical specificity [125]. Deep sequencing approaches, including duplex sequencing, allow detailed reconstruction of clonal evolution from a single sample and have proven particularly valuable in GB, where intratumoral heterogeneity and therapy-induced resistance are common [126,127,128,129,130]. Increasingly, NGS-based assays integrate epigenetic features and cfDNA fragmentation profiles, extending diagnostic capabilities beyond genomic alterations alone [131,132].

Nanopore sequencing has recently emerged as a complementary approach for cfDNA analysis. This long-read, amplification-free technology enables real-time interrogation of sequence variation, fragment length and DNA methylation on native DNA molecules [133]. These features are particularly relevant in CNS tumors, where ctDNA is scarce and characterized by tumor-specific fragmentation and methylation patterns. Recent studies demonstrate the feasibility of nanopore-based cfDNA profiling in brain tumors, including detection of copy number alterations and methylation signatures from plasma and CSF [134]. Notably, Vermeulen et al. showed that rapid nanopore sequencing combined with machine learning models can generate sparse methylation profiles of CNS tumors intraoperatively, highlighting its potential for real-time molecular diagnostics [135]. Although higher raw error rates currently limit its clinical deployment, continuous improvements in base-calling and bioinformatics pipelines are steadily enhancing performance [136,137].

DdPCR and NGS should be regarded as complementary rather than competing technologies. DdPCR offers maximal sensitivity and simplicity for targeted monitoring of predefined alterations and MRD, whereas NGS provides broader molecular coverage and enables detection of emerging resistance mechanisms and clonal diversity during therapy, albeit at higher cost and analytical complexity [138,139].

### 6.3. Not Only Molecular Biology

Recently, biophysical methods have complemented classical genetic analyses in LB, enabling assessment of molecular and structural properties of circulating biomarkers without amplification or sequencing. Raman spectroscopy detects characteristic molecular vibrations and can differentiate cancer patient samples from healthy individuals [140]. Infrared (IR) spectroscopy, particularly Fourier transform infrared spectroscopy (FT-IR), generates “biochemical fingerprints” of plasma or serum that reveal subtle differences associated with tumor progression, ctDNA presence, or changes in the tumor microenvironment [141,142,143]. Both techniques offer short analysis times and low material requirements. Differential scanning calorimetry (DSC) analyzes thermodynamic changes in plasma proteins, reflecting pathological modifications. DSC profiles differ between cancer patients and healthy individuals, linked to protein structural reorganization, immune complexes, and lipid fractions [144,145]. While DSC does not detect mutations, it provides unique biochemical information and complements genomic analyses. Overall, biophysical methods extend LB capabilities, enabling the detection of global biochemical changes at low cost and are useful for screening, patient stratification, and therapy monitoring.

### 6.4. Pre-Analytical Factors

Pre-analytical factors are crucial for the quality of cfDNA/ctDNA and for reliable results in ddPCR and NGS. Selecting appropriate blood collection tubes is essential: traditional EDTA tubes require immediate processing to prevent leukocyte lysis and gDNA contamination, which can dilute ctDNA and reduce sensitivity to low-allele mutations [146]. Stabilizing tubes (e.g., Streck BCT, PAXgene ccfDNA) preserve blood cells for 24–72 h, enabling transport without loss of integrity [147,148,149]. They are preferred when the processing time is unpredictable. The timing of the first centrifugation is also critical: EDTA tubes show a rapid increase in gDNA within 2 h, as confirmed in studies on processing delays [150,151]. In stabilizing tubes, this is delayed, but centrifugation within 6–24 h is recommended [152]. Transport and storage temperature further affect cfDNA quality, with higher temperatures accelerating leukocyte lysis. After plasma separation, material should be stored short-term at 4 °C and long-term at −80 °C, avoiding repeated freeze–thaw cycles that degrade fragments <150 bp [153]. High background cfDNA from leukocytes (>95% of plasma DNA) can reduce ctDNA detection, especially in low-proliferative tumors or after treatment [154,155]. Even the trim level of gDNA contamination lowers the ctDNA/gDNA ratio, increasing false negatives. Hemolysis introduces interfering substances, such as hemoglobin and free miRNA, which can disrupt PCR, RNA analyses, and cfDNA fragmentation assessment [156,157]. Optimized collection, stabilization, transport, and storage, combined with appropriate analytical methods, are essential for high-quality LB, particularly for subclonal mutation detection, MRD monitoring, and disease dynamics assessment. Standardized procedures and appropriate analytical technologies remain key to effective LB in precision oncology.

## 7. Scenarios Where Liquid Biopsy Can Change Clinical Practice

**Figure 2:**
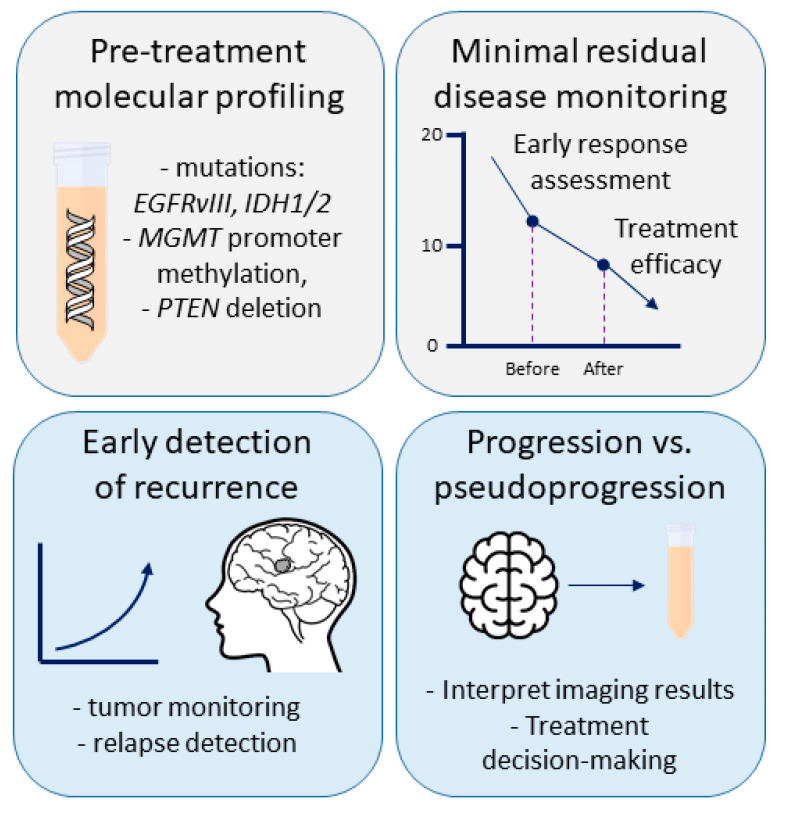
Clinical use cases of LB in GB. LB can support pre-treatment molecular profiling, monitoring of MRD, early detection of recurrence and differentiation between true progression (TP) and pseudoprogression (PsP), providing clinically relevant information complementary to neuroimaging and tissue biopsy.

LB is reshaping neuro-oncology by providing a non-invasive tool for dynamic disease monitoring, molecular profiling, and decision support when tissue access is difficult. In GB and CNS tumors, where biopsy is high-risk, LB may become part of routine practice. The following subsections illustrate scenarios where LB can impact clinical care (Figure 2).

### 7.1. Preoperative Diagnosis and Triage

Preoperative diagnosis of CNS tumors is challenging, as treatment decisions require rapid and reliable lesion assessment. When stereotactic biopsy is risky (e.g., brainstem tumors, medial lesions, or near eloquent structures), LB can aid triage. TriNetra-Glio isolates CTCs from peripheral blood using glial markers (GFAP, OLIG2) and mechanical properties for immunocytochemical verification. Proof-of-concept studies show CTCs are detectable in most high-grade glioma patients and virtually absent in healthy individuals, supporting LB in preoperative diagnostics [158,159]. While not yet a replacement for tissue biopsy, LB can reduce invasive procedures and assist treatment decisions in patients with poor general condition or tumor locations that prevent safe tissue collection.

### 7.2. Molecular Profiling Is Used When Tissue Is Limited or Risky to Obtain

A key element of modern neuro-oncology is precise molecular characterization, including mutations, copy-number alterations, amplifications, methylation profiles, and WHO 2021 classification [3]. In situations where access to tissue is limited or risky, an alternative is analysis of liquid material, primarily ctDNA and EV-DNA from CSF [3]. CSF testing is currently the most reliable LB method in CNS tumors, as the material circulates in direct contact with the tumor, bypassing the BBB that limits ctDNA in blood. Using NGS and epigenetic analyses, CSF-ctDNA reflects driver mutations such as EGFRvIII, *TERTp*, *TP53*, *PDGFRA*, and *IDH1*, while cfDNA methylation profiles allow glioma classification with accuracy similar to tissue-based methods, including molecular subtypes in metastatic tumors and DIPG (Diffuse Intrinsic Pontine Glioma) [110,160,161,162]. LB thus enables molecular analysis when tissue is scarce, degraded, contaminated, or unrepresentative due to spatial heterogeneity.

### 7.3. Monitoring Treatment Response, Resistance Mechanisms

Assessing treatment response in GB and other CNS tumors is challenging due to tumor heterogeneity, microenvironment variability, limitations of imaging, and therapy-induced inflammatory processes [163]. LB, including ctDNA, miRNA, EV cargo, and cfDNA fragmentation signatures, complements imaging and provides early molecular clues about disease activity. Dynamic changes in ctDNA level are particularly sensitive; rapid decreases after resection correlate with cytoreduction, while maintenance or renewed increases after chemoradiotherapy often precede radiological progression by weeks [27,30,164,165]. miRNA profiles, such as miR-21, miR-10b, and miR-221/222, reflect proliferation, invasiveness, and immune response, serving as stable indicators over time [166,167]. EVs offer more profound insight into resistance mechanisms, containing genetic and protein material reflecting cancer cell states, including PI3K/AKT, MET, and PDGFRA pathway changes, as well as epigenetic reprogramming linked to TMZ resistance. EV-lncRNA signatures, like HOTAIR, correlate with aggressive glioma phenotypes and increase during progression [168,169]. Combined analysis of ctDNA and EVs enables monitoring of tumor mass, clonal evolution, and activation of adaptive pathways, which are essential for targeted or immunotherapy [170,171].

### 7.4. Pseudoprogression and True Progression

Distinguishing TP from PsP is challenging, especially in the first 3–6 months after radiotherapy, when inflammation, BBB permeability, and peritumoral remodeling can mimic progression on MRI [163]. LB provides molecular markers for more accurate interpretation. In PsP, the ctDNA level is usually low or undetectable, reflecting the absence of active tumor proliferation. Stable or decreasing ctDNA combined with increasing radiological changes strongly suggests PsP [15,27]. Similarly, miR profiles remain stable, and EV signatures do not exhibit typical markers of invasiveness or angiogenesis; in some cases, EV-miRs linked to the immune response even increase, distinguishing PsP from TP [172]. In TP, molecular signatures change markedly. The ctDNA level rises weeks before MRI changes, while cfDNA fragmentation shifts toward short fragments <150 bp, indicating increased apoptosis of cancer cells [173,174]. EVs show increased lncRNA and protein expression associated with migration, angiogenesis, and therapeutic resistance, and increased activity of MET, EGFR, and PI3K/AKT pathways in EV cargo helps distinguish TP from PsP before imaging changes become apparent [175,176,177]. In recent years, integrating LB data with radiomics has become increasingly important. Combining quantitative imaging features—such as the relative cerebral blood volume (rCBV), apparent diffusion coefficient (ADC), Choline to N-acetylaspartate (NAA) ratio (Cho/NAA), diffusion, and perfusion parameters—with ctDNA, cfDNA fragmentation, and EV cargo enables predictive models with accuracy exceeding 85–90% [178,179]. Many radiology reviews emphasize that future diagnostic protocols will be based on hybrid algorithms combining MRI, LB, and AI analytics, thereby reducing ambiguous cases and limiting the need for additional biopsies [163,180].

### 7.5. Differential Diagnosis of Intracranial Lesions

LB may support the differential diagnosis of intracranial lesions with overlapping radiological features, a common challenge in neuro-oncology. GB often presents imaging characteristics similar to other malignant or inflammatory entities, including primary CNS lymphoma, metastatic brain disease, or treatment-related changes such as PsP and radiation necrosis [15,181]. Conventional MRI, even with perfusion imaging and diffusion-weighted sequences, may not reliably distinguish these pathologies. LB provides complementary molecular information reflecting tumor-specific biology. ctDNA carrying GB-specific alterations such as *EGFR* amplification or mutation, *TERT* promoter mutations, or *CDKN2A/B* loss can increase confidence in a GB diagnosis when detected in plasma or CSF. In contrast, the absence of these alterations or the presence of markers typical of other entities may prompt reconsideration of the initial diagnosis [110,112,113]. Beyond DNA-based markers, EVs enriched in GB-associated transcripts or proteins, including EGFRvIII, stemness-related RNAs, and immune-modulatory molecules like PD-L1, provide additional signals not visible on imaging [102,171,182]. Circulating RNA species, including selected miR signatures, also differ between GB and other intracranial tumors, supporting their use as adjunctive markers [183].

Importantly, LB cannot replace histopathological confirmation, especially at initial diagnosis, and should not be interpreted in isolation. Its adjunctive use may help narrow the differential diagnosis in selected scenarios, potentially reducing unnecessary repeat biopsies or guiding more appropriate diagnostic pathways. As LB technologies mature and analytical sensitivity improves, integrating molecular data with imaging and clinical context may enhance diagnostic confidence, facilitate earlier recognition of TP, and support more informed clinical decision-making in patients with suspected GB [9,15,184].

## 8. Evidence Level, Clinical Trials, and Regulatory Landscape

The development of LB in GB is mainly in exploratory studies and early clinical phases, with evidence still lower than for hematopoietic malignancies or lung cancer [23]. Most data come from case series, small single-center cohorts, and prospective observational studies in which LB is used alongside standard diagnostics, without directly influencing treatment decisions. These studies focus on detecting ctDNA and other biomarkers (miR, EVs, CTCs) in blood and CSF, and on their correlation with histopathological, molecular, and radiological features [185,186]. Ongoing and completed clinical trials show recurring patterns. First, pilot prospective observational studies (NCT04940507) collect blood and/or CSF before neurosurgery and at subsequent time points to assess the sensitivity and specificity of ctDNA, CTCs, or EVs relative to histopathology and MRI [187]. Second, prospective studies use LB as a monitoring tool, with serial sampling during chemoradiotherapy, recurrence treatment, or participation in targeted and immunotherapy trials (NCT06610682), analyzing ctDNA, methylation profiles, and EVs in relation to treatment response and early progression detection [164]. Third, some trials integrate LB into treatment protocols for patient stratification or study inclusion (e.g., eligibility for experimental therapies based on CSF ctDNA mutations). However, these remain conceptual and involve small patient groups (NCT06610682) [23]. Table 2 summarizes active and emerging trials investigating ctDNA, EV-derived markers, and circulating cells as LB tools in GB.

Most evidence in GB is level II–III (case series, small cohorts), with few larger prospective multicenter studies [3,23,186]. Randomized intervention studies in which clinical decisions are modified based on LB results are lacking, limiting the assessment of OS or PFS impact. Meta-analyses of LB with CSF have focused mainly on sensitivity, specificity, prognostic value, and feasibility, but full clinical utility remains unproven [24,188,189].

Guidelines from EANO, SNO, and NCCN consistently highlight the experimental nature of LB in brain tumors. EANO guidelines note CSF ctDNA as the most promising material but do not recommend routine use [3,190]. SNO guidelines emphasize diagnostic potential, response monitoring, resistance detection, and trial planning, but highlight the need for standardization and prospective evidence [27,189]. NCCN does not recommend LB as standard for GB; experiences in other malignancies are cited as a model for potential development [24,189].

For LB to be included in the SNO, EANO, or NCCN guidelines as standard care for GB, several conditions must be met. First, extensive prospective multicenter studies are needed in clearly defined clinical scenarios (e.g., PsP differentiation, treatment changes, targeted therapy eligibility) to evaluate the impact on outcomes [191]. Second, standardization of pre-analytical and analytical procedures is essential, including tube type, turnaround time, ctDNA/EV isolation, and validated cut-offs [35,192]. Third, regulators and payers require cost-effectiveness data showing LB adds value beyond histopathology and advanced imaging. Finally, tests must have regulatory approval (IVD/FDA/CE) specifically for GB, not only pan-cancer panels [24,189].

Currently, LB is at an advanced stage of biomarker validation and early clinical trials, with consensus on its potential but insufficient evidence for routine recommendations. The field is moving from feasibility and correlation studies toward clinical utility demonstration, where LB may become a pillar of adaptive, molecularly guided glioma treatment strategies.

## 9. Integration with Imaging, Radiomics, and AI/ML

Advances in imaging, radiomics, and ML enable synergistic use of LB and imaging in neuro-oncology, improving diagnostic accuracy, predicting treatment response, and detecting recurrence early, particularly in GB, where traditional assessments often prove insufficient.

Radiomics, analyzing textures, shapes, intensity, and spatial MRI features, provides insight into tumor heterogeneity, angiogenesis, proliferation, and microenvironment changes. Combining radiomics with LB biomarkers, ctDNA, cfDNA fragmentation, miRNA, and EV cargo yields predictive models superior to either alone [171]. Low ctDNA after radiotherapy, consistent with PsP radiomic signals (low rCBV, high ADC), allows confident classification of non-progressive lesions. In contrast, increased ctDNA with perfusion rises and decreased ADC indicate TP [178,179]. Integration with EVs-miRNA and lncRNA further improves early recurrence prediction, especially post-TMZ [102,193].

ML-based models combining radiomic perfusion/diffusion, EVs, and ctDNA signatures can predict chemoradiotherapy or immunotherapy responses weeks before scheduled MRI [178,179]. A dynamic ctDNA profile with radiomics can distinguish molecular progression from apparent stabilization (known as “molecular surrogate response”), potentially guiding adaptive therapy or trial enrollment [27,194].

ML allows complex integration of liquid biomarkers, MRI, clinical variables, and molecular tumor data. Algorithms such as gradient boosting, neural networks, and multidimensional supervised learning detect patterns invisible to classical statistics [195]. Models combining ctDNA, EVs (lncRNA/proteome), miR panels, and radiomics achieve high accuracy in distinguishing PsP from TP and detecting recurrence, with longitudinal ML further enhancing prognostic value [196].

Despite advances, several limitations prevent the full implementation of integrated ML models in routine practice. A key challenge is the small patient numbers in cohorts, especially when combining imaging and LB, which requires parallel sampling. Small samples can lead to overfitting, particularly in deep learning with many parameters [196]. External validation is limited; most models are tested at a single institution using the same patients or MRI equipment. Lack of standardization of pre-analytical and analytical procedures complicates comparisons across centers and reproducibility. Heterogeneity in imaging, including differences in MRI sequences, device settings, and operator variability, also disrupts the stability of radiomic features [21,28].

Developing integrated LB-radiomics-AI/ML platforms requires prospective multicenter studies and standardized “multi-omics + MRI” repositories. Nevertheless, the trend toward combining LB and advanced imaging is clear, and these technologies have the potential to become a key component of future diagnostic and prognostic algorithms in GB and other CNS cancers.

## 10. Challenges, Standardization, and Implementation

Despite the rapid development of LB and growing evidence for its potential in diagnosing and monitoring GB, several biological, technical, and organizational challenges limit its full clinical implementation. GB’s complexity affects the reliability of biomarkers from blood and CSF. The main issue is the BBB, which reduces tumor-derived ctDNA in peripheral circulation. GB also shows extreme intratumoral and spatial heterogeneity; different regions may have distinct mutations, methylation, and transcriptional profiles [30]. An important unresolved question in the field of LB is to what extent the analytes detected in biofluids accurately represent this heterogeneity, including whether all tumor compartments contribute. Small ctDNA amounts and predominance of cfDNA from host cells make low-allele mutation detection and clonal evolution monitoring technically challenging [62,185].

At the same time, serious technical challenges remain. LB for brain tumors lacks uniform standards across the diagnostic chain: pre-analytical procedures, analytical methods, and interpretation. Pre-analysis factors, such as tube choice, centrifugation, storage, and DNA or EV isolation, are unharmonized, and differences between centers can produce divergent results even with the same technologies [192]. There is no consensus on minimum quality requirements, including detection limits, error correction in sequencing, EV quality control, or miR standardization. Interpretation also lacks uniform cutoffs for ctDNA, cfDNA fragmentation, or EV-lncRNA signatures, often relying on researcher experience rather than validated clinical standards [35,149,197].

Implementing LB in clinical practice requires a systematic approach. Multicenter, prospective studies are essential to verify reproducibility across patient populations, centers, and equipment. Protocol harmonization must cover laboratory standards, material collection/processing, MRI sequence standardization for radiomics, and data reporting formats. These steps ensure comparability of results and support the creation of predictive models. Cost and clinical effectiveness analyses should demonstrate that LB improves care quality and provides economic value by reducing invasive biopsies, optimizing treatment, and identifying progression earlier [23,186].

Focused ultrasound-mediated BBB opening (FUS-BBBO) may partially address biological limitations by transiently increasing BBB permeability, enhancing the release of tumor-derived ctDNA, RNA, and EVs [198,199,200]. However, integrating FUS-BBBO with LB introduces challenges, including precise spatial targeting, standardization of ultrasound parameters, timing of blood sampling, and safety in repeated applications. FUS-BBBO is currently limited to select centers and investigational protocols, and its utility for routine monitoring is not established [201,202,203]. The representativeness of released biomarkers and their correlation with tumor burden or treatment response require further validation [200,204].

Only comprehensive harmonization, combined with demonstration of clinical and economic benefits, will allow LB to be included in SNO, EANO, and NCCN guidelines and to be fully implemented in routine neuro-oncology practice.

## 11. Ethical, Logistical, and Patient-Centered Aspects

Implementing LB in neuro-oncology requires attention not only to technological and clinical factors but also to ethical, logistical, and patient-centered issues. A key challenge is ensuring informed consent for molecular analyses, especially genomic sequencing and multi-omics testing. Patients must understand the scope of analyses, potential by-products (e.g., detection of germline variants), and rules for storing biological material [205,206]. With the growing role of AI/ML integrating clinical, imaging, and molecular data, enhanced privacy protection is needed through anonymization and controlled access to large datasets used for predictive algorithms [207,208].

From the patient’s perspective, LB is usually less burdensome than a classic tissue biopsy, but different forms vary in acceptability. Blood sampling, even serially, is generally well tolerated. CSF collection via lumbar puncture is more uncomfortable and carries a greater risk, so CSF-LB should be clinically justified, with benefits and risks carefully weighed [209].

Logistical factors also influence LB implementation: standardized procedures for sample collection, transport, and processing require adequate facilities, trained staff, and secure IT infrastructure. For dynamic monitoring, collection schedules must align with clinical decision points to maximize value [210,211,212].

## 12. Future Directions and Conclusions

LB has emerged as a rapidly advancing field in neuro-oncology, with the potential to complement and, in selected clinical scenarios, enhance conventional diagnostic and monitoring strategies in GB. Across analytes, including ctDNA, EVs, tumor-associated RNAs, and CTCs, LB captures molecular alterations and dynamic biological changes not accessible through imaging or single-site biopsy. Nevertheless, clinical integration remains limited due to technical, biological, and methodological challenges [62,191].

CSF-derived LB currently provides the most informative molecular readout, owing to its close anatomical proximity to the tumor microenvironment and its ability to capture key genetic alterations with high sensitivity. Nevertheless, the invasiveness and clinical constraints associated with lumbar puncture limit its applicability [213]. Plasma-based assays are minimally invasive and suitable for longitudinal monitoring, but the tumor signal remains low due to limited BBB efflux [109,214]. EVs are particularly promising: their lipid bilayer protects nucleic acids and proteins, reflecting tumor biology. EV-associated miRNA/RNA and EV-mediated mechanisms of resistance and immune evasion highlight their diagnostic and predictive potential [215,216]. Circulating miR, lncRNA, and circRNA provide complementary transcriptional information, while CTCs offer an additional, though less frequent, window into tumor phenotype [217].

First, there is a need for large, prospective, multicenter studies with harmonized protocols; however, clinical use may still be feasible without full interlaboratory standardization, provided that test–retest variability remains low [210,211]. Second, future research should prioritize panels of biomarkers rather than isolated analytes, as multi-parametric signatures provide greater robustness and biological relevance [72,186,191]. Third, integrating LB data with advanced neuroimaging, radiomics, and AI/ML offers a promising avenue to improve diagnostic accuracy, particularly for distinguishing TP from PsP [178].

Clinically meaningful use cases must be developed in which LB addresses unmet needs in GB care, including pre-treatment molecular profiling when tissue sampling is unsafe, monitoring MRD, and early detection of recurrence, and guiding therapy for actionable molecular targets or resistance mechanisms [83,218,219]. As LB becomes more incorporated into trials, its role in patient stratification and dynamic response assessment will expand [186,220].

In conclusion, while LB cannot yet replace surgical tissue diagnosis, it holds promise as a complementary tool to augment molecular characterization, refine disease monitoring, and provide mechanistic insights into treatment resistance and tumor-immune interactions. Continued advances in biomarker discovery, assay sensitivity, standardization, and integration with computational approaches are essential to unlock LB’s full potential in GB. With coordinated efforts, LB may become an integral component of personalized neuro-oncology.

## Figures and Tables

**Table 1:** Comparison of biofluids used for liquid biopsy in Glioblastoma (GB).

Biofluid	Molecular Yield	Detection Sensitivity	Advantages	Limitations	Bibliography
Cerebrospinal fluid	Highest (high variant allele frequency (VAF), ctDNA, abundant EV-RNA, low background cfDNA)	High (most sensitive biofluid; detects GB alterations in >60–70%)	Tumor-proximal; best representation of heterogeneity; high signal-to-noise; suitable for genomic profiling and clonal evolution	Invasive sampling; not feasible in all patients; requires neuraxial access	[21]
Plasma	Low–moderate (low VAF, diluted by systemic cfDNA)	Low–moderate (~20–40% depending on assay)	Minimally invasive; ideal for longitudinal monitoring; widely accessible; repeatable	BBB limits release of tumor DNA/EV; high background cfDNA; poor sensitivity for GB vs systemic tumors	[27]
Serum	Moderate (similar to plasma but higher background noise)	Low–moderate	Simple collection; can be paired with plasma comparative studies	Clotting-related release of genomic DNA from leukocytes; reduced ctDNA specificity	[23,70]
Urine	Very low (detectable EV-RNA; cfDNA extremely diluted)	Low	Non-invasive; patient-friendly; potential for home sampling	Very low tumor signal; still exploratory; needs ultrasensitive assays	[71]
Tumor-proximal venous blood	Moderate–high	Low–moderate	Higher concentration of tumor DNA than peripheral plasma; promising emerging method	Procedural complexity; limited clinical validation	[70]

**Table 2:** Selected ongoing and recent clinical trials involving liquid biopsy in glioma/glioblastoma (https://clinicaltrials.gov/, accessed on 25 November 2025).

ClinicalTrials.gov ID	Short Study Title	Population	Biofluid/Analyte	Study Focus	LB Objectives
NCT05925218	Circulating Tumor DNA Collection From Patients With High Grade Glioma or Other Brain Tumors	High-grade glioma/brain tumors	Blood/ctDNA	Observational collection of blood samples	Characterize ctDNA from high-grade glioma and other brain tumors as a basis for LB–based monitoring and diagnosis
NCT05934630	Testing Cerebrospinal Fluid for Cell-free Tumor DNA in Brain Tumor Patients	Brain tumor patients (including glioma/ GB)	CSF and blood/ cfDNA and ctDNA	Diagnostic/feasibility	Assess feasibility and sensitivity of detecting tumor cfDNA in CSF (and blood) as a non-invasive tool for brain tumor profiling
NCT05383872	Blood-Brain Barrier Disruption for Liquid Biopsy in Subjects With Glioblastoma Brain Tumors	Suspected or confirmed GB	Blood/ctDNA after BBB disruption	Interventional (BBB opening, diagnostic)	Evaluate whether ultrasound-mediated BBB disruption improves yield of blood-based LBin GB
NCT05133154	LIQUID BIOPSY IN Low-grade Glioma Patients	Low-grade glioma (with potential extension to higher grade)	Blood and CSF/ multiple markers	Prospective observational	Determine the value of blood/CSF LB as non-invasive, disease-associated biomarkers in glioma
NCT05964153	Analysis of Circulating DNA in Blood Samples of Glioma Patients	Glioma (various grades)	Blood/ ctDNA and CFDNA	Pilot diagnostic study	Investigate a new strategy for glioma diagnosis using circulating DNA-based LB protocol
NCT04940507 (BRAINFUL)	BRAINFUL: Brain Tumor Liquid Biopsy Stud	GB and other brain tumors	Blood and CSF/ multiple markers	Prospective, multi-modal biomarker study	Explore LB signatures in brain tumors; assess impact on GB management and prognostication
NCT04539431	Glioma Brain Tumours: Tissue and Liquid Biopsy Collection	Glioma/GB	Blood and CSF/ liquid biopsy	Prospective cohort with intraoperative sampling	Collect and analyze tumor tissue and matched LB (blood, CSF) obtained during surgery to correlate LB with tissue genomics
NCT04692324	Cerebrospinal Fluid Biomarkers for Brain Tumors	Primary metastatic brain tumors (including GB)	CSF/ multiple markers	Observational biomarker study	Identify and validate CSF biomarkers, including tumor-derived DNA and proteins, for diagnosis and monitoring of brain tumors
NCT05864534	Phase 2a Immune Modulation With Ultrasound for Newly Diagnosed Glioblastoma	Newly diagnosed GB	Blood/ctDNA (pre/post sonication)	Interventional (FUS + ICI) with LB correlative arm	Collect serial ctDNA to correlate with focused ultrasound–induced BBB modulation, treatment response and disease evolution

## Data Availability

No new data were created or analyzed in this study.

## Abbreviations

The following abbreviations are used in this manuscript:

ADC	Apparent Diffusion Coefficient
AI	Artificial Intelligence
APC	Adenomatous Polyposis Coli
BBB	Blood–Brain Barrier
CDKN2A/B	Cyclin-Dependent Kinase Inhibitor 2A and 2B
cfDNA	Cell-Free DNA
cfRNA	Cell-Free RNA
Cho/NAA	Choline (Cho) to N-acetylaspartate (NAA) ratio
circRNA	Circular RNA
CSF	Cerebrospinal Fluid
CTCs	Circulating Tumor Cells
ctDNA	Circulating Tumor DNA
ddPCR	Digital Droplet PCR
DIPG	Diffuse Intrinsic Pontine Glioma
EANO	European Association of Neuro-Oncology
EBM	Evidence Based Medicine
EGFR	Epidermal Growth Factor Receptor
EGFRvIII	Epidermal Growth Factor Receptor variant III
ERBB2	ERB-B2 Receptor Tyrosine Kinase 2
EVs	Extracellular Vesicles
FA	Fractional anisotropy
FUS-BBBO	Focused Ultrasound-Mediated Blood–Brain Barrier Opening
FT-IR	Fourier Transform Infrared Spectroscopy
GB	Glioblastoma
GFAP	Glial Fibrillary Acidic Protein
HOTAIR	HOX Transcript Antisense RNA
IDH1	Isocitrate Dehydrogenase 1
IL-13Rα2	Interleukin-13 Receptor subunit alpha-2
KDR	Kinase Insert Domain Receptor
LB	Liquid Biopsy
lncRNA	Long Noncoding RNA
LOD	Limit Of Detection
MET	Mesenchymal–Epithelial Transition Factor
ML	Machine Learning
miR	Micro RNA
MHC	Major Histocompatibility Complex
MGMT	Methylated-DNA–protein-cysteine methyltransferase
MRD	Minimal Residual Disease
MRI	Magnetic Resonance Imaging
NCCN	National Comprehensive Cancer Network
NF1	Neurofibromin 1
NGS	Next-Generation Sequencing
OR	Overall Response
PDGFRA	Platelet-Derived Growth Factor Receptor alpha
PD-L1	Programmed Death-Ligand 1
PET	Positron Emission Tomography
PFS	Progression Free Survival
PIK3CA	Phosphatidylinositol 3-Kinase Catalytic Subunit alpha
PsP	Pseudoprogression
PTEN	Phosphatase and Tensin Homolog
rCBV	Relative Cerebral Blood Volume
SNO	Society of Neuro-Oncology
TEPs	Tumor-Educated Platelets
TERT	Telomerase Reverse Transcriptase
TMZ	Temozolomide
TP	True Progression
TP53	Human Tumor Protein p53 gene
VAF	Variant Allele Frequency
